# Communicate to Care: Implementing Health Literacy in a Pediatric Ears, Nose, and Throat Clinic

**DOI:** 10.3928/24748307-20240819-02

**Published:** 2024-07

**Authors:** Carol J. Howe, Emily Carsey, Jordan Gamboa, Yan Zhang, Brennan Lewis

## Abstract

**Background:**

Despite positive outcomes in controlled trials, organizations have been slow to adopt health literacy practices. The purpose of the Communicate to CARE (Clear Communication, Achieve Understanding with Teach-Back, Receptive to our patient family needs, Empathetic care delivery) study was to use theories and strategies from implementation science to scale up health literacy practices in a pediatric Ears, Nose, and Throat (ENT) clinic.

**Brief Description of Activity:**

Expanding on previous efforts that simply reflected on barriers, the CARE team identified barriers within the local context pre-implementation to select strategies to directly address barriers during health literacy implementation. The RE-AIM framework was used to evaluate the reach, effectiveness, adoption, implementation, and maintenance of health literacy practices.

**Implementation:**

Over 18 months, the CARE team delivered multiple implementation strategies, including external facilitator, microlessons, preparing champions, audit and feedback, local consensus discussions, and small test of change. We tailored health literacy practices to clinic team roles to accommodate the clinic workflow.

**Results:**

ENT team mean ratings on acceptability, appropriateness, and feasibility remained >4 indicating a high likelihood of successful implementation. Caregiver *always* ratings significantly increased from baseline to 12 months for easy-to-understand medication instructions (74%–96%), test results (54%–96%), know what to do if had questions (89%–96%), and encouraged to talk about health problems (76%–90%). Caregiver ratings dropped slightly at 18 months, indicating a need for booster training. While one third of caregivers reported Teach-Back practice across all time periods, the ENT team reported increased practice from baseline (42%), 6 (61%) and 12 months (70%).

**Lessons Learned:**

Over the first 12 months, the external facilitator delivered implementation strategies with weekly contact, tapering contact over the final 6 months. The local champion became engaged in the CARE study through a quality improvement project with meaningful outcomes for the clinic and an incentivization program for scholarly endeavors. Lunch and learn sessions helped build relationships between the CARE and ENT team to discuss and problem solve issues. The 5-item CAHPS health literacy composite proved to be sensitive to changes during implementation of health literacy practices. Integrating these items into standard follow up surveys with patients and families would help realize the return on investment for health literacy implementation. [***HLRP: Health Literacy Research and Practice*. 2024;8(3):e166–e174.**]

Healthy People 2030 expanded the definition of health literacy beyond the individual person's capacities to include organizational health literacy, using policy to drive the roles and responsibilities of clinicians and organizations to make it easier for people to understand and use health information to take care of their health (Office of Disease Prevention and Health Promotion, n.d.). The call to action for the research community is to test interventions to improve organizational health literacy and measures to track its progress at the systems level (Santana et al., 2021). Early implementation efforts to integrate health literacy into practice may have failed because of contextual factors that worked against implementation in the real-world (DeWalt et al., 2011; Mabachi et al., 2016). Implementation science frameworks are helpful to identify pre-implementation barriers and facilitators, to inform choices of implementation strategies to address contextual factors, and to evaluate implementation effectiveness (Damschroder et al., 2022). Expanding on previous efforts, this study addressed critical gaps in our understanding of health literacy implementation in pediatric clinics.

## Purpose and Aims

The purpose of the Communicate to CARE study was to investigate the implementation of health literacy practices in pediatric ambulatory care. The specific aims were to (1) identify pre-implementation barriers and facilitators to select appropriate implementation strategies, and (2) evaluate the effectiveness of these strategies to enhance the uptake of health literacy practices.

### Background

One in three parents have low health literacy (Yin et al., 2009). Rudd (2007) reported that parents with less than high school education, less resources, and who were minority race/ethnicity or immigrants were at higher risk for lower health literacy than more educated, U.S. born, well-resourced parents. Low parent health literacy has been correlated with less health knowledge, confusion about medications, and increased preventable emergency room and 30-day readmissions with an estimated cost of $106 to $238 billion per year (Vernon et al., 2007).

The Agency for Healthcare Research and Quality (AHRQ) Health Literacy Universal Precautions (HLUP) toolkit guides practices to become health literate organizations (DeWalt et al., 2011). The toolkit includes 23 tools for action in 5 areas: Path to Improvement, Improve Spoken Communication, Improve Written Communication, Improve Self-Management and Empowerment, and Improve Supportive Systems (AHRQ, 2024). Several research teams have studied implementation of the toolkit. DeWalt et al. (2011) provided 8 practice-research networks a 4-month implementation plan for 5 HLUP tools over 8 weeks with 3 debriefing check points. Although the goal was to implement 5 tools, practices lacked resources, recommending a focus on 1 to 2 tools. Two months tested the tools but was not enough time to spread health literacy. Clinics viewed time constraints and the lack of facilitation as barriers. Mabachi et al. (2016) described implementation over 6 months in 12 primary care clinics. They provided technical assistance every 2 weeks and 2 site visits, using modest contact to determine what practices could do with minimal facilitation. Barriers included competing priorities, little quality improvement experience, and lack of leadership support. Gibson et al. (2022) used a health literacy training program with sustainability interventions in 25 primary care clinics. The team encouraged leaders to connect health literacy to institutional initiatives, distributed educational fliers, and provided lunch and learn sessions to practice health literacy. At 1 year, staff showed significantly improved knowledge, practice, and confidence with health literacy. Staff identified barriers, including time limits, uncertainty on how to use health literacy practices, and role constraints.

The complementary use of two implementation frameworks, CFIR (Consolidated Framework for Implementation Research) and RE-AIM (Reach, Effectiveness, Adoption, Implementation, Maintenance) offers an advantage in planning and evaluating health literacy implementation (King et al., 2020). CFIR provides a determinant framework to understand the drivers of effective implementation, breaking down barriers and facilitators across five domains: intervention characteristics, inner setting, outer setting, characteristics of individuals, and process. From this framework, an implementation strategy matching tool, CFIR-ERIC (Expert Recommendations for Implementing Change), emerges as a valuable resource. Powell et al. (2015) aligns 73 ERIC strategies with CFIR constructs, equipping implementers with strategies to meet challenges and optimize opportunities for advancing health literacy practices.

RE-AIM brings a practical lens to evaluating outcomes across five dimensions: Reach, Effectiveness, Adoption, Implementation, and Maintenance. Each dimension provides insights that guide us toward better health literacy practices.

## Description of Communicate to Care Study

### Research Design

This study used an exploratory, sequential mixed methods design. Phase 1 used qualitative methods (Howe et al., 2024), using CFIR to inform the interview guide, deductive coding, and data analysis (Damschroder et al., 2009). Interviews were conducted with stakeholders to identify barriers and facilitators. Barriers included compatibility of health literacy practices with the clinic workflow, staffing and time constraints, and the need for practice-based evidence, not research, for staff buy in. Facilitators included leadership support and the relative advantage of health literacy practices to meet the institution's mission to improve the patient experience. Identified barriers and facilitators were keyed into the CFIR-ERIC Matching tool to select implementation strategies (Powell et al., 2015) used in Phase 2 of the CARE study.

The purpose of this article is to report on the findings from Phase 2, which used a repeated cross-sectional design, measuring outcomes from both the ears, nose, and throat (ENT) team and parents over 18 months. This study was reviewed and approved by the University Institutional Review Board. The study team reviewed the purpose, benefits and risks of the study, addressed all questions, and obtained informed consent from participants.

### Setting and Sample

The setting was an ambulatory center at Children's Health, a large pediatric health system in Dallas, Texas. Children's Health operates 42 medical and surgical clinics with approximately 34,000 to 43,000 clinic visits per month, providing primary and specialty care to children. The ambulatory Associate Chief Nursing Officer invited all 42 clinics to participate in the CARE study. Potential participants included clinic teams and parents bringing their children for a clinic visit.

### Measures

The RE-AIM framework was used to evaluate health literacy practices (Holtrop et al., 2021), using specific measures to operationalize the RE-AIM dimensions (**Table 1**). The ENT team completed surveys at pre-implementation and at 6, 12, and 18 months. Surveys were conducted with parents at pre-implementation, 6 months, and 18 months post-implementation.

**Table 1:**
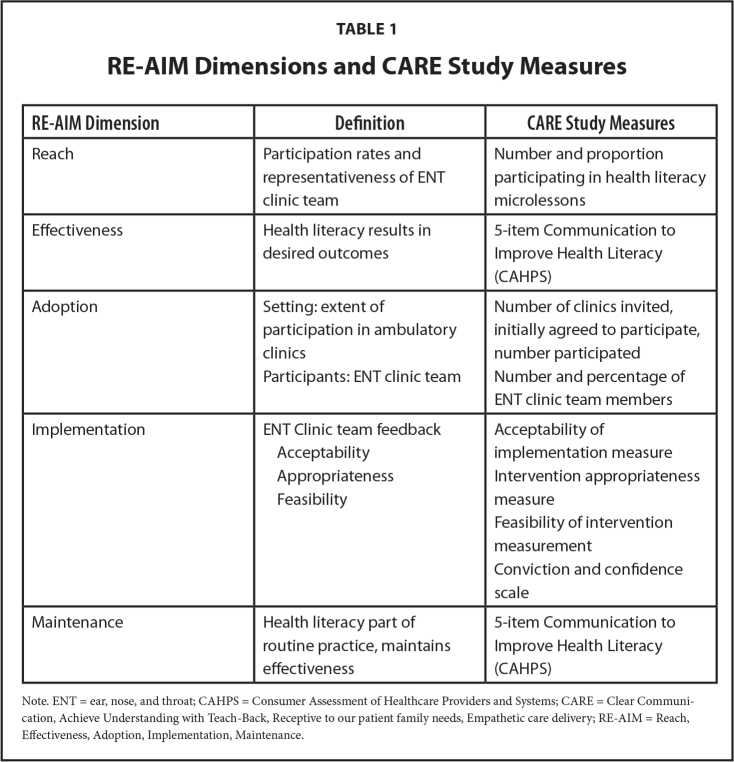
RE-AIM Dimensions and CARE Study Measures

**RE-AIM Dimension**	**Definition**	**CARE Study Measures**

Reach	Participation rates and representativeness of ENT clinic team	Number and proportion participating in health literacy microlessons

Effectiveness	Health literacy results in desired outcomes	5-item Communication to Improve Health Literacy (CAHPS)

Adoption	Setting: extent of participation in ambulatory clinics	Number of clinics invited, initially agreed to participate, number participated
	Participants: ENT clinic team	Number and percentage of ENT clinic team members

Implementation	ENT Clinic team feedback	Acceptability of implementation measure
	Acceptability	
	Appropriateness	Intervention appropriateness measure
	Feasibility	
		Feasibility of intervention measurement
		Conviction and confidence scale

Maintenance	Health literacy part of routine practice, maintains effectiveness	5-item Communication to Improve Health Literacy (CAHPS)

Note. ENT = ear, nose, and throat; CAHPS = Consumer Assessment of Healthcare Providers and Systems; CARE = Clear Communication, Achieve Understanding with Teach-Back, Receptive to our patient family needs, Empathetic care delivery; RE-AIM = Reach, Effectiveness, Adoption, Implementation, Maintenance.

***ENT team acceptability, appropriateness, feasibility.*** Acceptability of Implementation Measure assessed ENT team's belief that health literacy practices were agreeable, palatable, or satisfactory. Cronbach's alpha .85. Intervention Appropriateness Measure assessed reported perceived fit, relevance, or compatibility with health literacy practices in the clinic. Cronbach's alpha is .91. Feasibility of Intervention Measurement assessed belief that health literacy practices could be effectively carried out within their clinic. Cronbach's alpha is .89. Each measure included four items, that together take five minutes to complete and use a 5-point Likert scale from 1 = *completely disagree* to 5 = *completely agree*. Scores >4 indicated high likelihood of implementation success (Weiner et al. 2017).

***ENT team Conviction and Confidence Scale.*** The ENT team rated their use and confidence with health literacy practices on the Conviction and Confidence Scale. Four items ask about the use of Teach Back, using a 10-point Likert scale with higher scores indicating more conviction or confidence with Teach Back. Other items included intent to adopt Teach Back and use of plain language, avoiding *yes/no* questions, and using user friendly print materials. No psychometric data were available for Conviction and Confidence Scale although it has been used in earlier health literacy training (Feinberg et al., 2019). The scale takes less than 3 minutes to complete.

***Parent Communication to Improve Health Literacy of the Consumer Assessment of Healthcare Providers and Systems (CAHPS).*** This 5-item health literacy composite, available in English and Spanish, was used for parents to rate how well their providers addressed their health literacy needs (Weidmer et al., 2012). Participants responded on a 4-point Likert scale from 0 = *never* to 3 = *always* on items such as “How often did the provider give you easy to understand instructions on how to take your medicines?” and “How often did the provider ask you to repeat back or describe how you were going to follow the doctor's instructions?” Scores range from 0 to 15 with higher scores indicating better provider communication. The 5 item CAHPS demonstrated strong validity and reliability with reported Cronbach's alpha is .79.

## Description of Implementation Strategies

The study team included an academic nurse scientist, the Vice President of Nursing Excellence, both with expertise in health literacy, a biostatistician, and health literacy champions. We began with strategies identified in Phase 1: external facilitator, education, champions, audit & feedback, consensus discussions, and small tests of change. Overtime, the study team added strategy 8 to adapt health literacy practices into the clinic workflow and strategy 9 to leverage institutional incentives for champions to engage in the CARE study.

### Strategy 1. Engage an External Facilitator

The external facilitator (EF) was the nurse scientist who actively facilitated implementation strategies, including microlessons, champion development, and audit and feedback observations. During the first 12 months, the EF carried out implementation strategies in the ENT clinic for 2 to 3 hours weekly, tapering down to biweekly for 3 months and then monthly over the final 3 months. Although the EF initially drove implementation, these efforts were transitioned to the health literacy champions over time for sustainability.

### Strategy 2. Conduct Educational Outreach

The EF used a visual flipchart to deliver 5-minute microlessons during natural breaks in the clinic and the lunch hour. Microlessons focused on using plain language, limiting information to 3 to 5 key points, chunking information into manageable sections, and using Teach Back. Further training for nurses on the use of Teach Back included a slide presentation and role play of clinic specific scenarios. The EF addressed nurses' feelings of “testing” families, re-framing Teach Back as a communication technique that put the burden of explanation on clinicians. Examples of Teach Back questions were shared for nurses to adapt into their own language.

### Strategy 3. Identify and Prepare Champions

Two ENT nurses were appointed by clinic leadership as champions with little initial knowledge of the role responsibilities. Although reluctant at first, the lead champion evolved to be a key member of the study team. The lead champion helped deliver the microlessons, facilitated data collection, organized meetings, sent weekly emails to the ENT team to share health literacy tips and modeled health literacy practices. The second champion was unable to continue due to role constraints. A third clinic nurse with previous health literacy experience volunteered to become a champion 12 months into the study.

### Strategy 4. Audit & Feedback

The EF completed audits using the AHRQ Communication Observation form to monitor the interactions between a parent and clinicians. Items include, for example, “Did this clinician explain things in a way that was easy to understand?” and “Did the clinician ask the patient to describe how they were going to follow these instructions?”. The EF indicated yes/no and included comments to help the clinician improve their communication. The EF provided aggregate feedback to the ENT team through emails and infographics and used these observation times to coach and model health literacy practices.

### Strategy 5: Conduct Local Consensus Discussions

Lunch and learn sessions provided opportunities for the study and ENT teams to discuss what was working or not working, to problem-solve, and to build relationships.

### Strategy 6: Conduct Cyclical Small Tests of Change

The nurse champion coached by the study team led a quality improvement (QI) project to improve patient education for tonsillectomy and adenoidectomy surgery. The goal was to decrease preventable emergency department visits. The QI project included small tests of change focused on health literacy practices, including a sample pain management schedule, videos to reinforce care instructions, and a text-messaging program to share post operative care reminders at key intervals.

### Strategy 7: Obtain and Use Patient Feedback

The study team collected parent ratings on the CAHPS health literacy survey.

### Strategy 8: Promote Adaptability

The study team mapped and optimized the clinic workflow by tailoring role-based health literacy practices. The EF noted that the ENT team each had their “clinical script,” reciting treatment options, obtaining surgical consent, and/or providing patient education. Delivered in one long sentence, providers did not chunk information making it difficult to discern when they moved from one topic to another. The EF coached providers to provide “signposts like headings in a manuscript” to parents on the topics they were covering: “I need to go over several things with you… first, your child's study results… second, I want to go over treatment options… and last I want to talk about the risks and benefits of surgery.” Similarly, nurses provided extensive patient education but realized that parents did not retain information due to trends in post operative calls. The study team helped nurses prioritize their patient teaching to 3–5 “need to know” points and to use chunk and check with Teach Back. The “nice to know” information remained in the patient handouts.

### Strategy 9: Incentive Structures

The health system's clinical nurse achievement program motivated the champions. They earned points, which translated to a monetary bonus, for submission of abstracts, presentations, and papers. The lead champion also integrated health literacy practices into annual performance appraisals.

## Data Analysis

Data analysis was conducted using IBM SPSS Statistics (Version 29; IBM Corp., 2022) and JASP (Version 18; JASP Team, 2023). Descriptive statistics were employed to describe the characteristics of the ENT team and the proportion of ENT team participating in microlessons. These statistics also quantified mean ratings of acceptability, appropriateness, and feasibility and the proportion of self-reported health literacy practices across four time points.

For the parent data, descriptive statistics were used to characterize the sample. To assess item frequencies in CAHPS scores at 3 time periods, pre-implementation, at 6 months, and 18 months, crosstabulation and Chi-square tests were employed. A CAHPS sum score (global rating) was derived by summing the scores from all five CAHPS items. Correlations were conducted to assess the internal consistency of the CAHPS items (i.e., the CAHPS sum score vs individual items). Pearson's correlation was used when assumptions for parametric tests were met, and Spearman's rho was employed when these assumptions were not met.

## Results

### Reach

Of a total of 58 ENT team members, 39 (67%) participated in health literacy microlessons. The proportion of ENT team participation in microlessons by clinic role included 76% of providers (physicians and advanced practice providers), 87.5% of nurses, 80% of medical assistants and schedulers, and 100% of clinic leadership.

### Effectiveness

A convenience sample of 588 parents completed the CAHPS survey across three time points: pre-implementation (*n* = 196), at 12 months (*n* = 198), and at 18 months (*n* = 194). Parents were invited by a research assistant to verbally complete the survey in either English or Spanish immediately after their clinic visit. Most parents were age 25 to 49 years (83%), with 47% Hispanic, 29% White, 16% Black or African American, 5% Asian, and 3% biracial. Education levels included 22% high school, 34% Bachelor's, and 8% graduate degree.

The Robust Welch test and subsequent post-hoc analysis revealed a significant increase in mean CAHPS sum scores from pre-implementation (*M* = 12.36, standard deviation [*SD*] = 2.30) to 12 months (*M* = 13.00, *SD* = 1.73, *p* = .016). However, there were no significant changes from 12 months to 18 months or from baseline to 18 months.

At 12 months, parents indicated more frequent instances of health literacy practices. Most reported that providers offered easily understandable test results (96.2%) and provided clear instructions about medicines (95.7%), encouraged them to engage in more meaningful conversations (90.3%) and supplied all the information they needed (89.3%). These improvements were notable when compared to pre-implementation. At 18 months, slight drops in health literacy practice ratings were evident although these ratings remained above baseline. The only CAHPS item that remained consistently low across the three data collection periods was providers asking patients to repeat back their instructions [Teach Back].

Regarding internal consistency, Spearman's rho correlations revealed that CAHPS sum scores were significantly and strongly related when providers asked patients to repeat back (*r* = .807), while demonstrating moderate correlations with the remaining CAHPS items (**Table 2**).

**Table 2:**
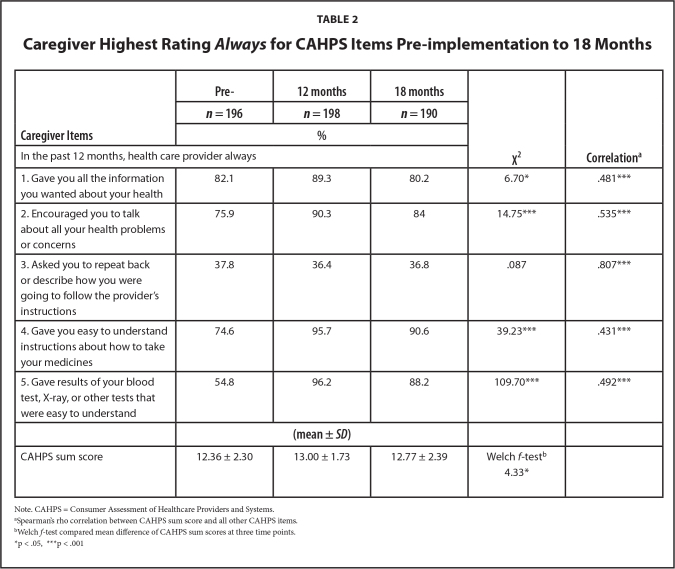
Caregiver Highest Rating *Always* for CAHPS Items Pre-implementation to 18 Months

**Caregiver Items**	**Pre-**	**12 months**	**18 months**	χ**^2^**	**Correlation^a^**
	***n* = 196**	***n* = 198**	***n* = 190**		
	**%**				
In the past 12 months, health care provider always					
1. Gave you all the information you wanted about your health	82.1	89.3	80.2	6.70*	.481***
2. Encouraged you to talk about all your health problems or concerns	75.9	90.3	84	14.75***	.535***
3. Asked you to repeat back or describe how you were going to follow the provider's instructions	37.8	36.4	36.8	.087	.807***
4. Gave you easy to understand instructions about how to take your medicines	74.6	95.7	90.6	39.23***	.431***
5. Gave results of your blood test, X-ray, or other tests that were easy to understand	54.8	96.2	88.2	109.70***	.492***
	**(mean ± *SD*)**				
CAHPS sum score	12.36 ± 2.30	13.00 ± 1.73	12.77 ± 2.39	Welch *f*-test^b^4.33*	

Note. CAHPS = Consumer Assessment of Healthcare Providers and Systems.

a Spearman's rho correlation between CAHPS sum score and all other CAHPS items.

b Welch *f*-test compared mean difference of CAHPS sum scores at three time points.

* p < .05,

*** p < .001

### Adoption

Although all 42 clinics were invited to participate in the CARE study, only three clinics, ENT, Sickle Cell, and Diabetes Clinics, responded to the call. However, once internal funding was secured, the Sickle Cell and Diabetes clinics declined participation due to short staffing and time constraints post–COVID-19. The ENT Clinic agreed to participate with support from the Division Chief and Practice Administrator. This was a busy clinic, seeing up to 130 patient visits per day in 15-minute appointments. The ENT team provided patient education about clinical test and laboratory results, options for surgical and medical treatment, and post-operative care instructions.

### Implementation

The clinic team rated acceptability, appropriateness, and feasibility of health literacy practices >4 across all time periods, indicating a high likelihood of successful implementation.

**Figure 1.**
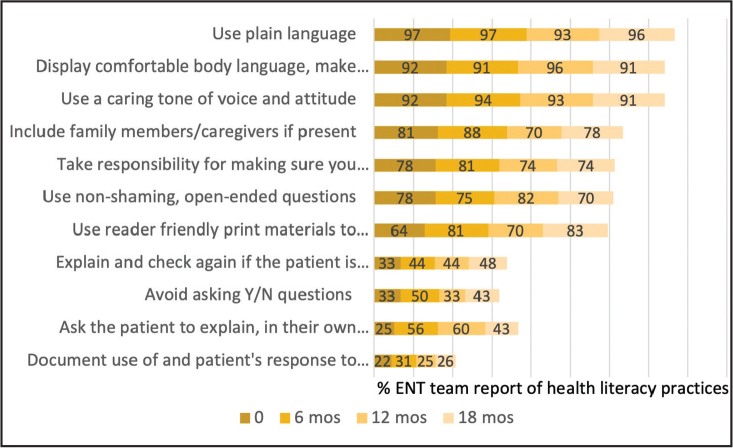
Ears, nose, and throat reports of health literacy practices at baseline—6, 12, and 18 months.

Mean ratings for conviction that Teach Back was important remained consistent (8.2–8.3/10) across the time periods, while mean confidence with Teach Back increased from 6.7 at baseline to 7.8 at 18 months. The proportion of the clinic team who reported using Teach Back steadily increased from 41.7% pre-implementation to 75% at 18 months (**Figure 1**).

## Lessons Learned

By connecting the CARE study purpose of scaling up health literacy practices to the organizational mission of improving the patient experience, the ENT leadership welcomed the participation in the CARE study. Although we had envisioned several clinics participating in our study, we learned that one participating clinic was optimum given our resources of personnel, materials, and time. We offer lessons learned about the study team, implementation strategies, and outcomes with the hopes that our insights will be useful for other implementation teams.

### The Study Team

The external facilitator and internal champions drove intensive implementation that leveraged the health literacy and implementation expertise of EF and the knowledge of the local clinic context of the champions. The success of dual facilitator roles to influence successful implementation of health literacy practices has been reported previously (Kaper et al., 2021).

Ayre et al. (2023) highlighted the role of an internal champion to drive implementation. In this study, the champions evolved to be key members of the study team. Initially, an appointed health literacy lead champion was reluctant, confirming previous reports that appointed versus volunteer champions were less likely to be enthusiastically involved (Kaper et al., 2021; Mabachi et al., 2016). The change in mind-set of the lead champion pivoted at approximately 9 months into the CARE study after the VP of Nursing Excellence engaged the champions in a QI project to reduce preventable emergency department visits within 30 days of tonsillectomy and adenoidectomy surgery, a meaningful outcome to the ENT team. The success of this QI effort increased buy in from the ENT team, validating recent suggestions that implementation science and QI methods should be aligned rather than considered disparate approaches to implementation (Leeman et al., 2021).

### Implementation Strategies

The study team deployed implementation strategies over 18 months. In contrast, earlier studies used a 2-month (DeWalt et al., 2011) and 6-month (Mabachi et al., 2016) implementation period that failed to realize sustained health literacy practices. More recently, (Gibson et al., 2022) provided implementation efforts over 15 months, showing significant changes in health literacy practices at 12 months with a slight dip in practices at 15 months. Similarly, in this study, improvements in health literacy were observed at 12 months with a slight dip at 18 months, indicating the need for “boosters” to continually hardwire practice change.

Throughout the first year, the EF provided implementation strategies weekly in the ENT clinic (Alagoz et al., 2018). In contrast, previous studies reported less direct contact with clinics, ranging from check-ins at project milestones (DeWalt et al., 2011) to every two weeks for technical assistance (Mabachi et al., 2016). Successful implementation in this study suggested that a higher “dose” of an EF may be needed for successful implementation of health literacy practices, confirming the time and effort of this role (Kaper et al., 2021).

The study team accommodated time constraints of the ENT team with microlessons and lunch and learns. These strategies provided efficient, creative approaches the study team used to share and connect with the ENT team. These connections uncovered opportunities to adapt different aspects of health literacy practices for different team members within the clinic workflow, which may have influenced the high acceptability, appropriateness, and feasibility scores.

The ENT team became accustomed to the EF audits and were responsive to EF coaching and modeling of health literacy practices. Early on, clinic staff requested real-time feedback but quickly became defensive with any suggestions for improvement. In response, the EF created aggregate feedback in friendly infographics and emails, highlighting the ENT team's strengths with gentle suggestions for improvement (Landis-Lewis et al., 2020). Although the champions volunteered to assume audit and feedback, conflicting responsibilities prevented them from completing routine observations, confirming previous findings of the time constraints of champions (Brach, 2017; Howe et al., 2020).

### Outcomes

The CARE study adds to the literature on implementation of health literacy by including patient reports which have been scarce (Kaper et al., 2021). In this study, the 5-item health literacy CAHPS composite proved sensitive to changes in health literacy practices (Weidmer et al., 2012). Significant increases in parent reports of health literacy practices at 12 months provided practice-based outcomes that validated the CARE study in the minds of the ENT team which proved to be a crucial turning point for the support of health literacy practices. A research assistant captured CAPHS surveys at the end of clinic which may not be feasible without research funding. Although organizations may be wary to add survey items, advocating to include health literacy composite items in vendor supplied patient experience surveys would provide data on the return on investment for health literacy implementation projects.

The ENT team reported increasing use of health literacy practices, with 70% reporting use of Teach Back at the end of the study. In contrast, parent reports of experiencing Teach Back remained steady at 36–37% across all time periods. The disparate results between the ENT team self-report and parent ratings may reveal social desirability bias. Similarly, Gibson et al. (2022) reported no change with 26% of the primary care team reporting Teach Back post-training; 70%, however, reported that they had corrected misunderstandings with their patients which posed an interesting and maybe better way to capture the intent of the Teach-Back technique.

## Study Limitations

This study has several limitations. Because this study took place in one clinic with no control clinic, these findings are not generalizable. The repeated cross-sectional design of this study offered population-level changes over time, but it did not capture individual-level changes, limiting insights into causal relationships. Future research could benefit from a mixed-methods approach, incorporating cross-sectional and cohort elements to provide longitudinal data, balancing feasibility and depth of analysis in clinical settings. Additionally, future studies may consider outcomes of interest to clinical teams, including decreases in triage phone calls, preventable emergency department visits, and 30-day admissions; these outcomes would contribute to our understanding of the effectiveness of health literacy practices in real-world settings.

## Implications

The CARE study team implementation efforts were substantial. Future studies, informed by implementation science, are needed to discover the implementation “dose” to integrate health literacy practices into clinician-patient interactions. Although no clinics except the ENT clinic signed onto the CARE study, the success of the study team has now reached into the organization. The champions developed a virtual learning community and are now leading a health literacy implementation project in 24 additional clinics. The study team expanded and embarked on a new health literacy implementation study informed by the iPARIHS framework. We know from the CARE study and that by Gibson et al. (2022) that there was a downward drift in health literacy practices over time, suggesting the need for at least a 12-month implementation period with subsequent booster trainings.

Future studies should use direct observation or recordings of clinical interactions to determine the fidelity to health literacy practices. Although the AHRQ communication observation rubric was helpful for audit and feedback, further testing and adaptation of this communication observation tool would add rigor to fidelity evaluation of clinician health literacy practices.
